# Magnetic resonance imaging arterial spin labeling hypoperfusion with diffusion-weighted image hyperintensity is useful for diagnostic imaging of Creutzfeldt–Jakob disease

**DOI:** 10.3389/fneur.2023.1242615

**Published:** 2023-10-10

**Authors:** Yuki Kitazaki, Masamichi Ikawa, Tadanori Hamano, Hirohito Sasaki, Tomohisa Yamaguchi, Soichi Enomoto, Norimichi Shirafuji, Kouji Hayashi, Osamu Yamamura, Tetsuya Tsujikawa, Hidehiko Okazawa, Hirohiko Kimura, Yasunari Nakamoto

**Affiliations:** ^1^Second Department of Internal Medicine, Faculty of Medical Sciences, University of Fukui, Fukui, Japan; ^2^Biomedical Imaging Research Center, University of Fukui, Fukui, Japan; ^3^Department of Advanced Medicine for Community Healthcare, Faculty of Medical Sciences, University of Fukui, Fukui, Japan; ^4^Department of Aging and Dementia (DAD), University of Fukui, Fukui, Japan; ^5^Life Science Innovation Center, University of Fukui, Fukui, Japan; ^6^Department of Rehabilitation, Faculty of Health Science, Fukui Health Science University, Fukui, Japan; ^7^Department of Radiology, Faculty of Medical Sciences, University of Fukui, Fukui, Japan

**Keywords:** arterial spin labeling, cerebral blood flow, magnetic resonance imaging, Creutzfeldt–Jakob disease, epilepsy

## Abstract

**Background and objectives:**

Magnetic resonance imaging with arterial spin labeling (ASL) perfusion imaging is a noninvasive method for quantifying cerebral blood flow (CBF). We aimed to evaluate the clinical utility of ASL perfusion imaging to aid in the diagnosis of Creutzfeldt–Jakob disease (CJD).

**Methods:**

This retrospective study enrolled 10 clinically diagnosed with probable sporadic CJD (sCJD) based on the National CJD Research & Surveillance Unit and EuroCJD criteria and 18 healthy controls (HCs). Diffusion-weighted images (DWIs), CBF images obtained from ASL, *N*-isopropyl-(^123^I)-*p*-iodoamphetamine (^123^IMP)-single-photon emission computed tomography (SPECT) images, and ^18^F-fluorodeoxyglucose (^18^FDG)-positron emission tomography (PET) images were analyzed. First, the cortical values obtained using volume-of-interest (VOI) analysis were normalized using the global mean in each modality. The cortical regions were classified into DWI-High (≥ +1 SD) and DWI-Normal (< +1 SD) regions according to the DWI-intensity values. The normalized cortical values were compared between the two regions for each modality. Second, each modality value was defined as ASL hypoperfusion (< −1 SD), SPECT hypoperfusion (< −1 SD), and PET low accumulation (< −1 SD). The overall agreement rate of DWIs with ASL-CBF, SPECT, and PET was calculated. Third, regression analyses between the normalized ASL-CBF values and normalized SPECT or PET values derived from the VOIs were performed using a scatter plot.

**Results:**

The mean values of ASL-CBF (*N* = 10), ^123^IMP-SPECT (*N* = 8), and ^18^FDG-PET (*N* = 3) in DWI-High regions were significantly lower than those in the DWI-Normal regions (*p* < 0.001 for all); however, HCs (*N* = 18) showed no significant differences in ASL-CBF between the two regions. The overall agreement rate of DWI (high or normal) with ASL-CBF (hypoperfusion or normal) (81.8%) was similar to that of SPECT (85.2%) and PET (78.5%) in CJD. The regression analysis showed that the normalized ASL-CBF values significantly correlated with the normalized SPECT (*r* = 0.44, *p* < 0.001) and PET values (*r* = 0.46, *p* < 0.001) in CJD.

**Discussion:**

Patients with CJD showed ASL hypoperfusion in lesions with DWI hyperintensity, suggesting that ASL-CBF could be beneficial for the diagnostic aid of CJD.

## 1. Introduction

Creutzfeldt–Jakob disease (CJD) is the most common form of transmissible spongiform encephalopathy that includes idiopathic (sporadic), genetic (inherited), and acquired (infectious) disorders. CJD, particularly the sporadic type (sCJD), typically presents with rapidly progressive dementia, leading to serious impairment of the patients’ quality of life (1). Thus, accurate and early diagnosis of CJD is critically important for patient self-determination and infection control.

The clinical diagnosis of sCJD is based on characteristic neurological symptoms, such as progressive cognitive decline and myoclonus, and periodic synchronous discharges (PSDs) on electroencephalography (EEG), as well as extremely high tau protein and positive 14–3-3 protein levels in the cerebrospinal fluid (CSF). In addition, the detection of abnormal prion protein levels using the real-time quake (RT-QUIC) method is almost certainly indicative of sCJD (2–4). However, the early neurological manifestations of sCJD, such as mild cognitive impairment, are often nonspecific. Furthermore, PSDs on EEG usually manifest in the advanced stage of sCJD, and high tau protein and positive 14-3-3 protein levels in the CSF can be detected in other neurological diseases (5, 6). As a result, examination of abnormal prion protein levels in the CSF is required in patients who present with partial clinical findings, which delays the diagnosis of sCJD (7).

Diffusion-weighted images (DWIs) derived from magnetic resonance imaging (MRI) are a useful tool in establishing a diagnosis of CJD; specifically, in patients with sCJD, DWIs usually show regional hyperintensity in the striatum, cortical gray matter, and thalamus (7, 8). Although cortical hyperintensities observed on DWIs have a high sensitivity for the early clinical diagnosis of CJD (9), they are sometimes difficult to distinguish from artifacts, epilepsy, and encephalitis (e.g., herpes simplex virus encephalitis and autoimmune encephalitis [AE]), which may show similar DWI findings (10, 11).

Several studies from the past two decades using single-photon emission computed tomography (SPECT) or positron emission tomography (PET) have revealed a reduction in the regional cerebral blood flow (CBF) or glucose metabolism in patients with CJD (12–15). Furthermore, Xing et al. reported that hyperintense lesions on DWIs were approximately consistent with regions showing decreased glucose metabolism in the PET images of patients with CJD (16). Although SPECT and PET may be useful for the diagnostic imaging of CJD, both modalities have some disadvantages, such as relatively higher costs, radiation exposure, difficult repeatability, and institutional limitations.

As an alternative to these radiopharmaceutical imaging techniques, MRI with arterial spin labeling (ASL) perfusion imaging has been developed as a noninvasive method for quantifying CBF (17). The advantages of ASL imaging include short scanning times, no requirement for contrast administration, and the feasibility of performing studies as part of standard MRI protocols, including DWIs. Therefore, ASL images have been widely used to evaluate cerebral perfusion changes in neurodegenerative and cerebrovascular diseases (18–20). However, the utility of ASL imaging as the diagnostic aid of CJD remains unclear.

To evaluate the clinical application of ASL imaging to aid in the diagnosis of CJD, we retrospectively investigated CBF in the cortical regions showing DWI hyperintensities using ASL images in patients with CJD, and compared ASL findings with SPECT and PET findings.

## 2. Materials and methods

### 2.1. Patients

We conducted a retrospective case-series study of 10 patients with CJD (6 men, mean age, 67.3 ± 8.1 years) who were classified as having probable sCJD based on the National CJD Research & Surveillance Unit (21) and EuroCJD (7) criteria for sCJD between January 2011 and May 2020 at the University of Fukui Hospital, Japan. The demographic and clinical characteristics of the patients with CJD are summarized in Table 1. None of the patients had a family or medical history of suspected iatrogenic CJD. All 10 patients presented with symptoms related to sCJD, such as myoclonus, pyramidal tract signs, extrapyramidal tract signs, or cerebellar ataxia, at the time of diagnosis. In addition, all patients showed PSDs on EEG and positive 14-3-3 protein and RT-QuIC results in their CSF. Genetic testing for the prion gene was performed in four of the 10 patients, and none showed pathogenic mutations (Table 1).

**Table 1:** Demographic and clinical characteristics of the patients with Creutzfeldt–Jakob disease (CJD).

CJD patient #	CJD 1	CJD 2	CJD 3	CJD 4	CJD 5	CJD 6	CJD 7	CJD 8	CJD 9	CJD 10
Age (1st MRI)	70s	50s	80s	70s	60s	70s	60s	60s	50s	60s
Codon129	Met/Met	Met/Met	Met/Met	N/A	N/A	Met/Met	N/A	N/A	N/A	N/A
Codon219	Glu/Glu	Glu/Glu	Glu/Glu	N/A	N/A	Glu/Glu	N/A	N/A	N/A	N/A
Mutations of PrP gene	−	−	−	N/A	N/A	−	N/A	N/A	N/A	N/A
Initial clinical symptoms										
Myoclonus	+	−	−	+	−	−	−	+	−	−
Visual disturbance	+	−	+	+	−	−	+	+	−	+
Pyramidal tract sign	+	+	+	−	+	+	−	+	+	+
Extrapyramidal tract sign	+	−	+	+	+	−	+	+	−	+
Cerebellar ataxia	+	+	+	−	+	+	+	−	+	−
Akinetic mutism	−	−	−	−	−	−	+	−	−	−
Laboratory findings										
PSDs in EEG	+	+	+	+	+	+	+	+	+	+
14–3-3 protein in CSF	+	+	+	+	+	+	+	+	+	+
RT-QUIC in CSF	+	+	+	+	+	+	+	+	+	+
Duration from clinical onset (days)										
MRI	25	24	28	92	62	28	33	29	28	58
^123^IMP-SPECT	30	28	32	91	61	38	38	35	N/A	N/A
^18^FDG-PET	31	29	44	N/A	N/A	N/A	N/A	N/A	N/A	N/A

ASL, arterial spin labeling; CJD, Creutzfeldt–Jacob disease; CSF, cerebrospinal fluid; DWI, diffusion weighted image; EEG, electroencephalogram; F, Female; ^18^FDG-PET, ^18^F-fluorodeoxyglucose positron emission tomography; Glu, glutamine; ^123^IMP-SPECT, *N*-isopropyl-(^123^I)-*p*-iodoamphetamine single-photon emission computed tomography; M, male; Met, methionine; MRI, magnetic resonance imaging; N/A, not available; PrP, prion protein; PSDs, periodic synchronous discharges.

We also included the ASL-CBF data of 18 healthy controls (HCs) (nine men; average age, 68.6 ± 7.8 years) and three patients with AE (three men; mean age, 57.3 ± 6.8 years) who underwent brain MRI during the same period. These HCs showed no neurological defects or obvious MRI abnormalities. All patients with AE had no epileptic seizures during MRI imaging and showed positive serum results for anti-neuronal antibodies: two patients with gamma-aminobutyric acid B receptor (GABA_B_R) and one patient with leucine-rich glioma-inactivation 1 (LGI1) (22). There were no significant differences in age and sex between patients with CJD and HCs.

Brain MRI, including DWI and ASL sequences, was performed in all patients with CJD, HCs, and patients with AE. *N*-isopropyl-(^123^I)-*p*-iodoamphetamine (^123^IMP)-SPECT was performed in eight of the 10 patients (CJD 1–8), and^18^F-fluorodeoxyglucose (^18^FDG)-PET was performed in three of the 10 patients (CJD 1–3). The durations from the clinical onset of CJD to the initial study using each modality were 24–92 days for brain MRI, 28–102 days for ^123^IMP-SPECT, and 29–44 days for ^18^FDG-PET (Table 1).

This study was approved by the Institutional Review Board of the University of Fukui (20170130). The ethics committee waived the need for informed consent because of the retrospective nature of this study.

### 2.2. MRI acquisition

Brain MRI with DWI, T1-weighted, and ASL perfusion imaging was performed using a 3 T-MR clinical scanner (Discovery MR750 3.0 T; GE Healthcare, Milwaukee, WI, USA) as a routine clinical scan. DWIs were obtained using axial diffusion-weighted echo-planar sequences (*b* value = 1,000 s/mm^2^, TR/TE = 6000/66.9). A high-resolution three-dimensional T1-weighted anatomical image was acquired using the following parameters: repetition time (TR) = 7.2 ms; echo time (TE) = 2.2 ms; inversion time (TI) = 700 ms; flip angle = 10; field-of-view (FOV) = 240 mm; 512 × 512 matrix; 224 slices; and voxel dimension = 0.47 × 0.47 × 0.7 mm^3^. For ASL imaging, a pseudo-continuous ASL scheme was employed using the protocol as described in the previous studies (19, 20, 23–25). In brief, we prepared a three-dimensional spiral fast spin echo sequence with background suppression covering the entire brain. The acquisition parameters were as follows: seven arms with 512 points for each spiral arm; 34–40 slices; section thickness = 4 mm; TR = 6 s; labeling duration = 1.5 s; post-labeling delay (PLD) = 1.5 or 2.0 s; image reconstruction matrix = 128 × 128; and NEX = 3, as previously described (19, 20, 24, 25). Each PLD was set at 1.5 s from January 2011 to February 2015 and as 2.0 s from March 2015 to May 2020, and each subject was imaged with one type of PLD. Approximate proton-density-weighted images were acquired using the same acquisition parameters, except for TR = 2 s and with no background suppression. The T1 of arterial blood water and the whole-brain average blood/brain partition coefficient were set to 1.6 and 0.9 s, respectively, for CBF quantification. The number of patients with CJD who underwent ASL imaging with each PLD (1.5 s or 2.0 s) was two and eight, respectively; the corresponding number of HCs was five and 13, and the number of patients with AE was one and two, respectively.

### 2.3. SPECT imaging acquisition

All ^123^IMP-SPECT studies were performed using an integrated SPECT/CT system (Symbia T; Siemens Healthcare, Erlangen, Germany) with dual-head scanners and a LEHR collimator. The SPECT scans were performed 30 min after the intravenous injection of 222 MBq of ^123^IMP into all patients with CJD using routine clinical protocols. The SPECT image matrix and pixel size were 128 × 128 through 360° (180° for each head) rotation at 5° intervals, with 45 s per arc interval. We used a damping factor of 0.15 cm^−1^ based on previously obtained pooled phantom data. The scatter component of the projection data was corrected using the triple-energy window method. All image sets were reconstructed from the image data with CT attenuation correction and scatter correction.

### 2.4. PET imaging acquisition

Brain PET scans with ^18^FDG were performed using a whole-body combined PET/CT scanner (Biograph mCT; Siemens Healthcare, Erlangen, Germany). ^18^FDG was produced using an automated FDG synthesis system (26). The time coincidence window was 4.1 ns, the system time resolution was 540 ps, and the energy window was 435–650 keV. All procedures were performed according to routine clinical protocols, including fasting for at least 4 h prior to receiving an intravenous injection of 185 MBq ^18^FDG. The patients were immobilized on the PET/CT scanner 50 min after the injection to prevent head movement during imaging for CT attenuation correction. The reconstructed images were converted to standardized uptake value images of each patient’s body weight and injected dose.

### 2.5. Image processing

Quantitative image analysis of T1-weighted images, DWIs, ASL-CBF maps (in 10 patients with CJD, 18 HCs, and three patients with AE), and ^123^IMP-SPECT (in eight patients with CJD), and ^18^FDG-PET (in three patients with CJD) images was performed using PMOD 3.6 software (PMOD Technologies Ltd., Zurich, Switzerland). The transformation matrix was obtained by fusing the T1-based spatial normalization using the PNEURO tool in PMOD with the acquired images for each subject. All images were matched and spatially normalized using an individual transformation matrix. The images of the cerebral cortex were separated by segmentation and applied to a preset template of PMOD (Hammers N30R83 atlas) (27, 28). The resulting transformation matrix was used to transform the T1-based Hammers template, and all the images were spatially normalized. The Hammers atlas was adjusted to the T1-weighted images in a spatial normalization procedure to create an individual anatomical parcellation of volumes of interest (VOIs) (27, 28). After T1-based spatial normalization, 62 cortical regions were identified and converted to VOIs using the Hammers atlas. Regional cortical values derived from the obtained VOIs were normalized using the whole gray matter values in each imaging modality (i.e., DWI intensity, ASL-CBF, SPECT perfusion, and PET uptake values) of each subject.

### 2.6. Statistical analysis

All statistical analyses were performed using IBM SPSS Statistics for Windows, version 23.0 (IBM Corp., Armonk, NY, USA). Data are expressed as mean ± standard deviation (SD). Statistical significance was set at *p* < 0.05.

Sixty-two cortical regions were identified in each subject’s DWIs, and regional cortical values derived from the obtained VOIs were normalized using the whole gray matter values. We classified the cortical VOIs as a DWI-High (involved) or DWI-Normal (intact) region according to the SD of the normalized DWI values in each subject (≥ +1 SD and <+1 SD, respectively). Cutoff values were set to evaluate DWI-High numerically and consistently. Among the 62 VOIs established, the mean number of VOIs per group at ≥ +1 SD, defined as DWI-High regions, was calculated as 9.3 ± 1.3 for the CJD group, 8.3 ± 1.8 for the HC group, and 7.7 ± 2.1 for the AE group. Supplementary Image 1 shows the normalized DWI values in the 62 VOIs with the +1 SD line in each patient with CJD.

A two-tailed unpaired *t*-test was used to evaluate the differences in the normalized ASL-CBF, SPECT, and PET values between the DWI-High and DWI-Normal regions for each subject and each group, respectively. Bonferroni correction for multiple comparisons was used for the statistical analysis of differences in normalized ASL-CBF values among the CJD, HC, and AE groups in the DWI-High regions.

Regression analyses between the normalized ASL-CBF values and normalized SPECT or PET values derived from the VOIs were performed using a scatter plot with Pearson’s product–moment correlation coefficient (*r*) in patients with CJD.

We defined each modality value as ASL hypoperfusion (< −1 SD of the mean normalized ASL-CBF value of each subject), SPECT hypoperfusion (< −1 SD of the mean normalized SPECT value of each patient), and PET low accumulation (< −1 SD of the mean normalized PET value of each patient). The overall agreement rate between DWIs and ASL-CBF in patients with CJD was calculated using the following formula described previously by Kundel et al.: {(the number of regions showing both DWI-High and ASL hypoperfusion) + (the number of regions showing both DWI-Normal and ASL normoperfusion)}/(the total number of regions) (29). The overall agreement rates between DWIs and SPECT or PET were also calculated in the same manner. Cohen’s kappa coefficient (κ) with a 95% confidence interval (CI) was derived on the basis of the overall agreement rates, as per the study by McHugh (30).

## 3. Results

### 3.1. Representative images

**Figure 1:**
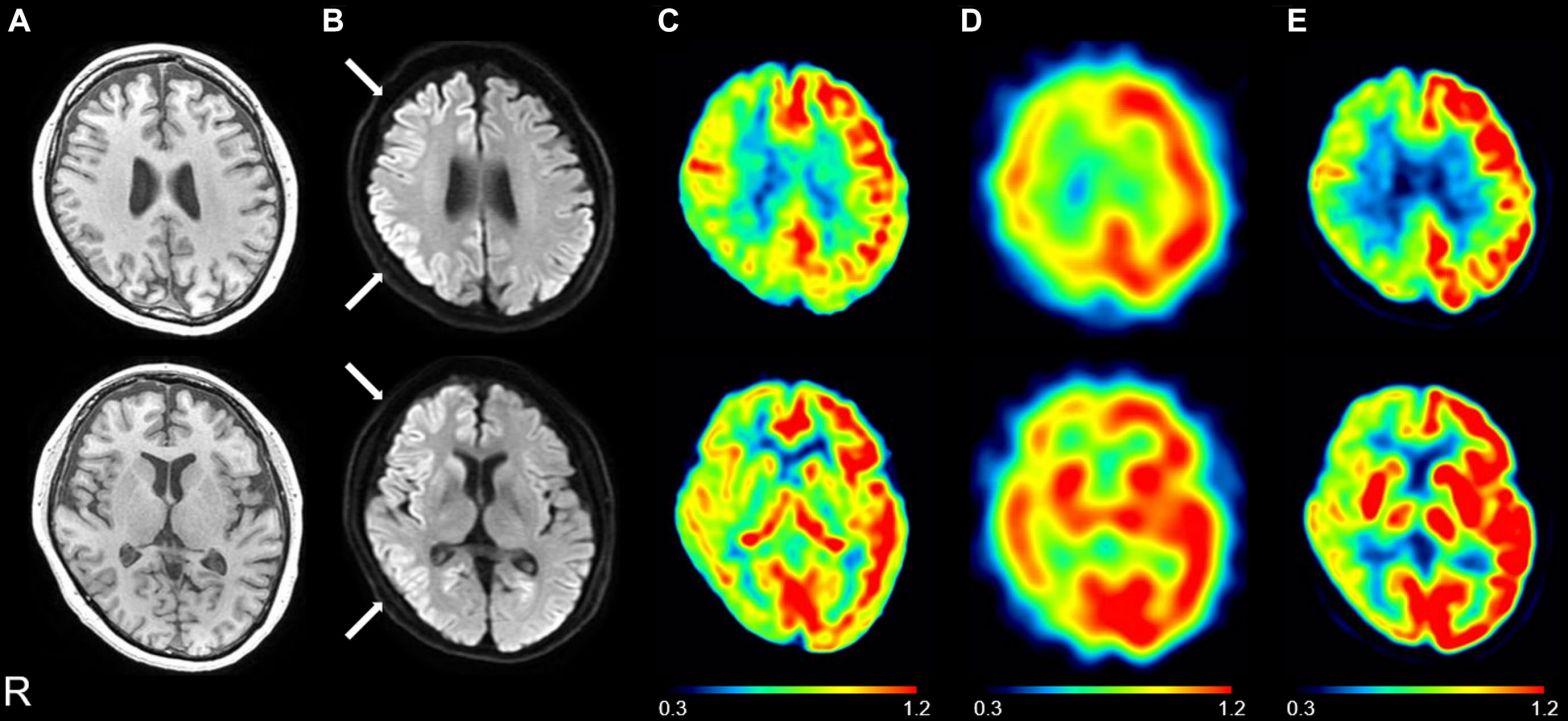
Representative images of a patient with Creutzfeldt–Jakob disease (CJD) (CJD 2: patient in their 50s). Brain magnetic resonance imaging showed normal findings on the T1-weighted image **(A)**; the right caudate nucleus, frontal, parietal, and temporal hyperintensity on the diffusion-weighted image **(B)** (see white arrows); and the right caudate nucleus, frontal, parietal, and temporal hypoperfusion on the cerebral blood flow map derived from arterial spin labeling (ASL) imaging **(C)** on day 24. ^123^I-*N*-isopropyl-*p*-iodoamphetamine single-photon emission computed tomography (SPECT) revealed right, frontal, parietal, and temporal hypoperfusion **(D)** on day 28, and ^18^F-fluorodeoxyglucose positron emission tomography (PET) revealed right caudate nucleus, frontal, parietal, and temporal hypoperfusion **(E)** on day 29. The color bar indicates the normalized values of ASL perfusion **(C)**, SPECT perfusion **(D)**, and PET uptake **(E)**.

Figure 1 shows the brain MR images, including T1-weighted images, DWIs, and ASL-CBF maps, as well as the ^123^IMP-SPECT and ^18^FDG-PET images of a representative patient with CJD (CJD 2, patient in their 50s). DWIs showed regional hyperintensity in the right caudate nucleus as well as the frontal, parietal, and temporal cortices. In contrast, the ASL-CBF, SPECT, and PET images exhibited hypoperfusion or low accumulation in these regions, suggesting that the ASL hypoperfusion regions were consistent with the DWI hyperintensity regions.

**Figure 2:**
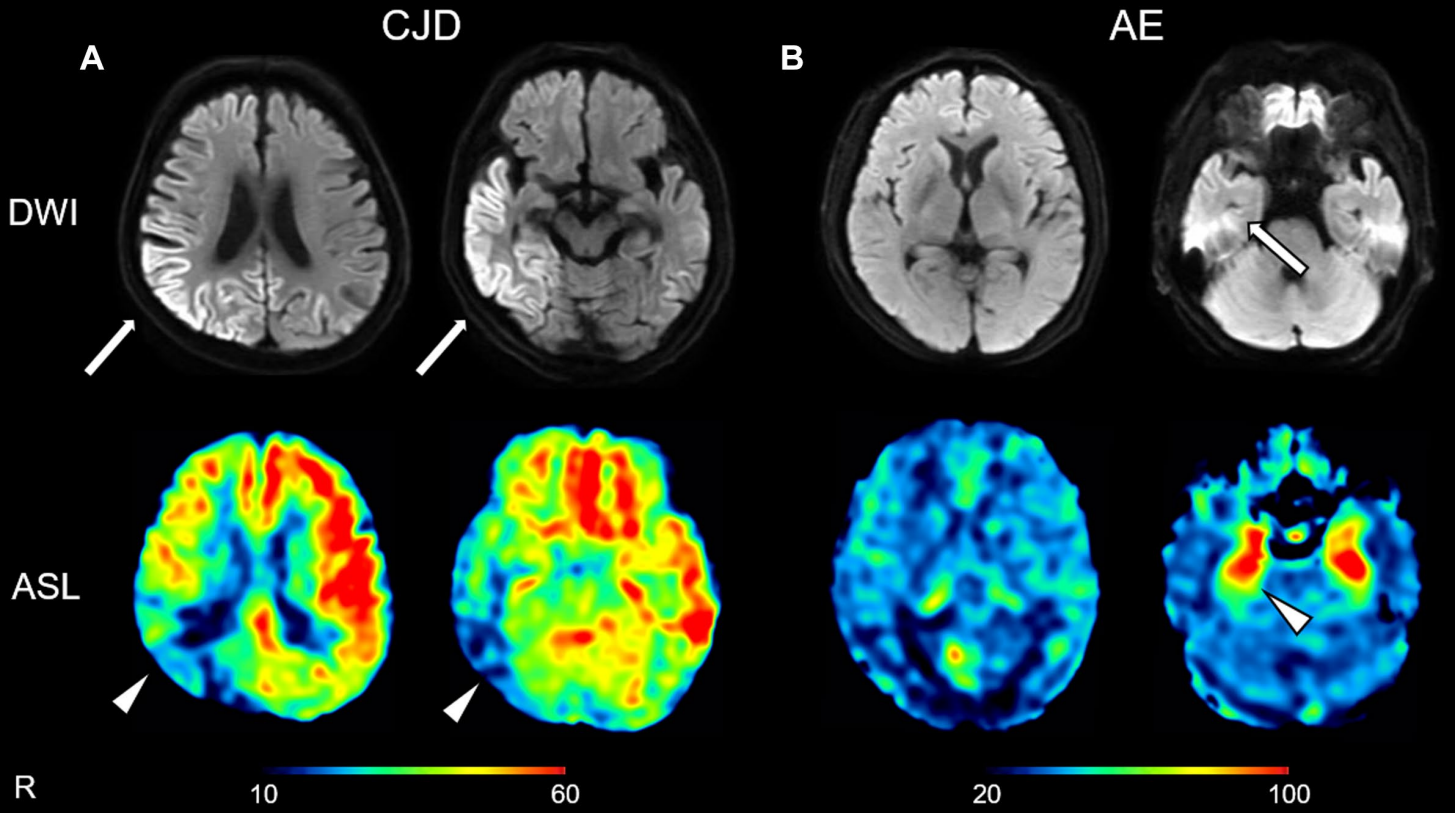
Representative diffusion-weighted images (DWIs) and cerebral blood flow maps derived from arterial spin labeling (ASL) imaging of a patient with Creutzfeldt–Jakob disease (CJD) (**A**: CJD 4, patient in their 70s) and a patient with autoimmune encephalitis (AE) associated with anti-leucine-rich glioma-inactivated 1 antibodies (**B**: AE 3, patient in their 60s). The patient with CJD exhibited DWI hyperintensity in the right parietal and temporal regions (arrows) that showed ASL hypoperfusion (arrowheads) **(A)**. The patient with AE exhibited DWI hyperintensity in the right temporal region (arrow) that showed ASL hyperperfusion (arrowhead) **(B)**. The color bars indicate the range of cerebral blood flow values (ml/100 mg/min) (**A,B**).

Figure 2 shows the brain MR images of another representative patient with CJD (CJD 4, patient in their 70s) and a representative patient with AE associated with anti-LGI1 antibodies (AE 3, patient in their 60s). The patient with CJD exhibited DWI hyperintensity in the right parietal and temporal regions that showed ASL hypoperfusion (Figure 2A). In contrast, the patient with AE exhibited DWI hyperintensity in the right temporal regions that showed ASL hyperperfusion (Figure 2B).

### 3.2. Values of each modality for the corresponding DWI values

**Figure 3:**
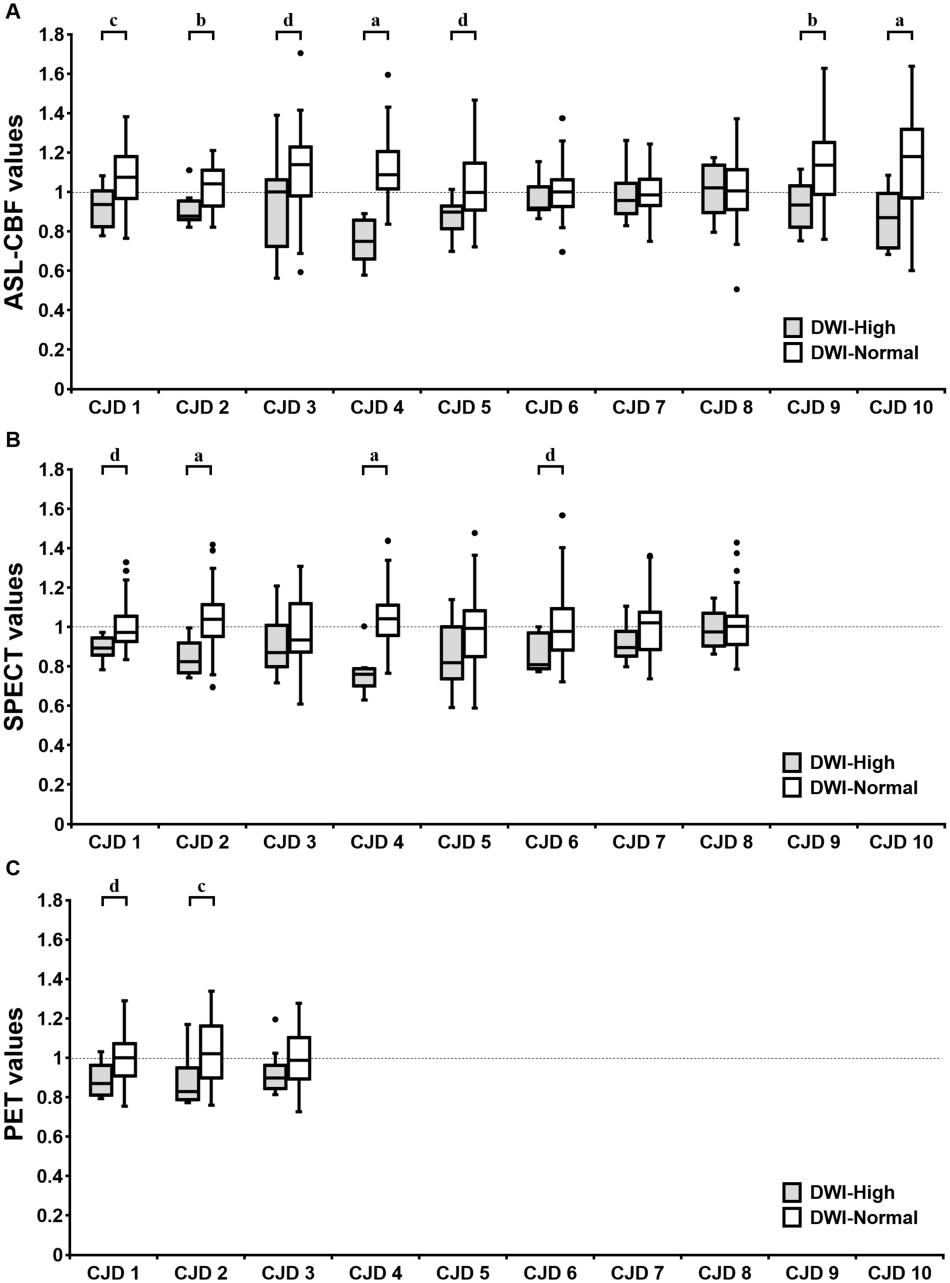
Box plot of the normalized values of arterial spin labeling (ASL)-cerebral blood flow **(A)**, single-photon emission computed tomography **(B)**, and positron emission tomography **(C)** in the DWI-High regions (gray box) and DWI-Normal regions (white box) in each patient with Creutzfeldt–Jakob disease (CJD) **(A–C)**. A *t*-test was used to evaluate the differences between the DWI-High and DWI-Normal regions. ^a^Value of *p* of *t*-test <0.001, ^b^Value of *p* of *t*-test <0.005, ^c^Value of *p* of *t*-test <0.01, ^d^Value of *p* of *t*-test <0.05, compared with DWI-Normal. DWI-H, DWI-High; DWI-N, DWI-Normal.

**Figure 4:**
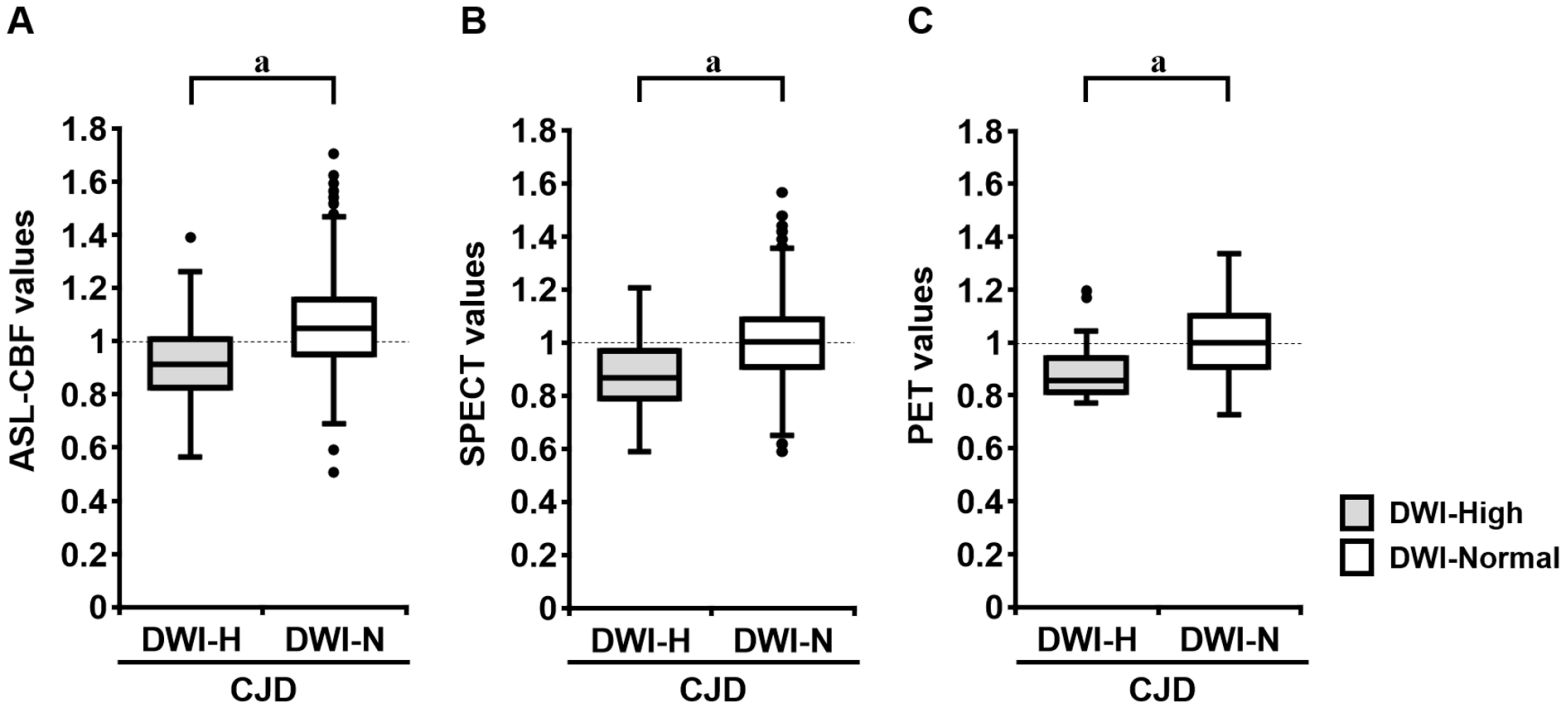
Box plot of the normalized values of arterial spin labeling (ASL)-cerebral blood flow (CBF) (*N* = 10; **A**), single-photon emission computed tomography (SPECT) (*N* = 8; **B**), and positron emission tomography (PET) (*N* = 3; **C**) in the DWI-High and DWI-Normal regions in patients with Creutzfeldt–Jakob disease (CJD). The mean ASL-CBF, SPECT, and PET values in the DWI-High regions were significantly reduced compared with those in the DWI-Normal regions (*p* < 0.001 for all) **(A–C)**. A *t*-test was used to evaluate the differences between the DWI-High and DWI-Normal regions. ^a^Value of *p* of *t*-test <0.001, compared with DWI-Normal.

The mean normalized ASL-CBF values in the DWI-High regions were significantly lower than those in the DWI-Normal regions in seven of the 10 patients with CJD (Figure 3). Similarly, four of the eight patients who underwent ^123^IMP-SPECT and two of the three patients who underwent ^18^FDG-PET showed significantly lower mean normalized VOI values in the DWI-High regions than in the DWI-Normal regions (Figure 3). The mean normalized values of ASL-CBF, SPECT, and PET were significantly decreased in the DWI-High regions compared with the DWI-Normal regions (*p* < 0.001 for all) (Figure 4) (Supplementary Table 1). In contrast, the mean normalized ASL-CBF values showed no differences between the DWI-High and DWI-Normal regions in HCs and patients with AE, for the subject and group levels (Supplementary Image 2).

**Figure 5:**
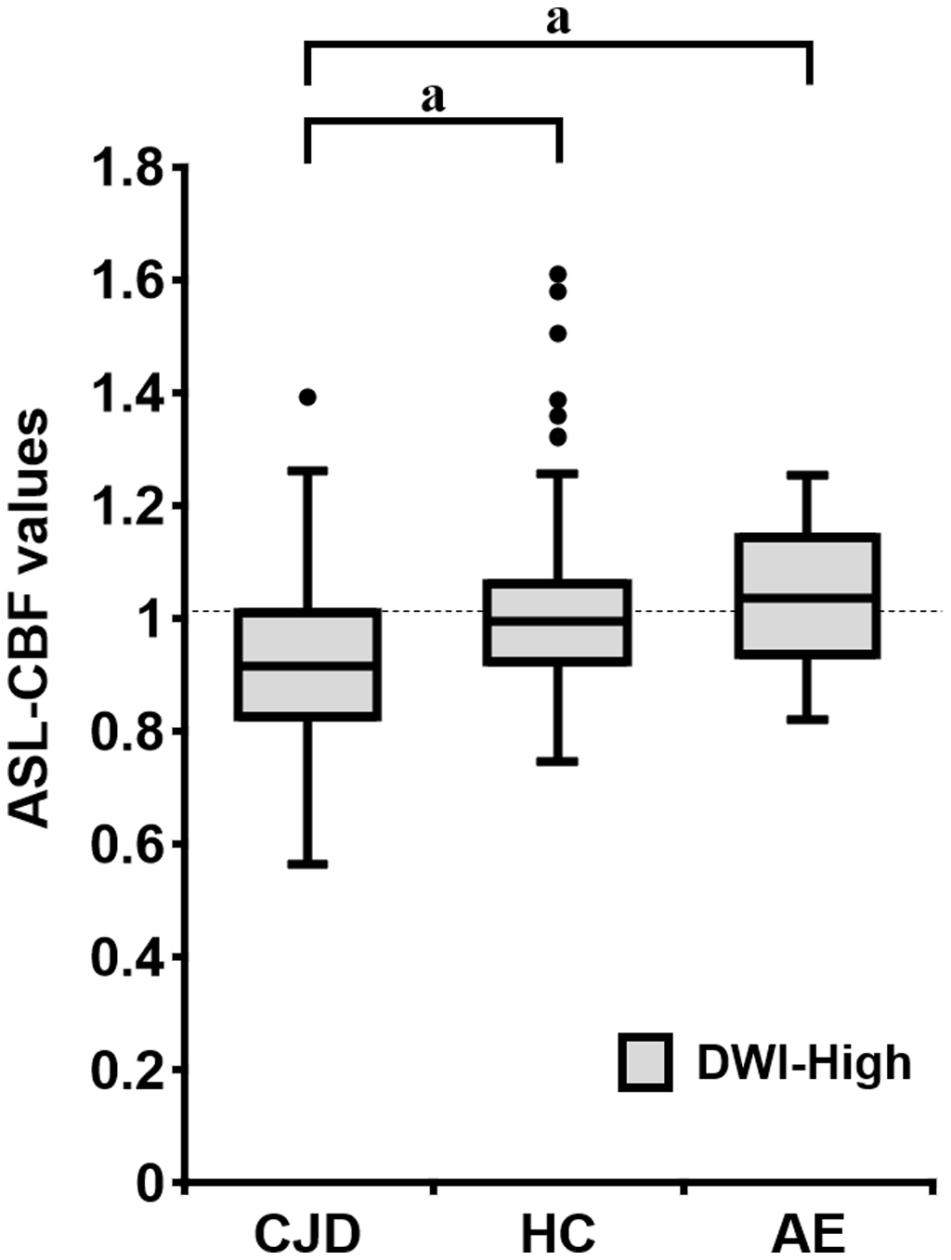
Box plots of the normalized arterial spin labeling (ASL)-cerebral blood flow (CBF) values of patients with Creutzfeldt–Jakob disease (CJD) (*N* = 10), HC subjects (*N* = 18), and patients with autoimmune encephalitis (AE) (*N* = 3) in the DWI-High region. The mean normalized ASL-CBF values of patients with CJD were significantly lower than those of HC subjects and patients with AE, for each (*p* < 0.001, respectively). Bonferroni correction for multiple comparisons were applied to evaluate the differences in the normalized ASL-CBF values. ^a^Value of *p* < 0.001, compared with patients with CJD.

In a group-by-group comparison among the patients with CJD, HCs, and patients with AE, the mean normalized ASL-CBF values of the patients with CJD were significantly lower than those of the HCs and patients with AE in the DWI-High regions, according to Bonferroni correction (*p* < 0.001, respectively) (Figure 5).

### 3.3. Agreement rates of each modality to DWI values

We compared the overall agreement rates and kappa statistics with the 95% CIs of DWI values in the same regions for ASL-CBF, ^123^IMP-SPECT, and ^18^FDG-PET in patients with CJD. ASL-CBF showed the overall agreement rate and kappa statistic of DWI values (81.8% and 0.29 [95% CI, 0.19–0.39], respectively), which were comparable with those of SPECT (85.2% and 0.37 [95% CI, 0.25–0.48], respectively) and PET (78.5% and 0.23 [95% CI, 0.06–0.40], respectively) (Table 2).

**Table 2:** The overall agreement rate between DWI and ASL-CBF values (*N* = 10), SPECT values (*N* = 8), and PET values (*N* = 3) was calculated using the following formula described previously by Kundel et al. (29) in all patients with CJD.

		DWI values		Overall agreement rate	Kappa statistic	95% CI
		High	Normal			
ASL-CBF values (*N* = 10)	Hypoperfusion	37 (6.0%)	58 (9.4%)	81.8%	0.29	0.19–0.39
	Normal	55 (8.9%)	490 (75.8%)			
SPECT values (*N* = 8)	Hypoperfusion	30 (6.0%)	40 (8.1%)	85.2%	0.37	0.25–0.48
	Normal	33 (6.7%)	393 (79.2%)			
PET values (*N* = 3)	Low accumulation	11 (5.9%)	25 (13.4%)	78.5%	0.23	0.06–0.40
	Normal	15 (8.1%)	135 (72.6%)			

Cohen’s kappa coefficient with a 95% confidence interval (CI) was derived based on the overall agreement rates. ASL, arterial spin labeling; CBF, cerebral blood flow; CI, confidence interval; CJD, Creutzfeldt–Jacob disease; DWI, diffusion–weighted image; PET, positron emission tomography; SPECT, single-photon emission computed tomography.

### 3.4. Correlations of ASL-CBF values to SPECT and PET values

**Figure 6:**
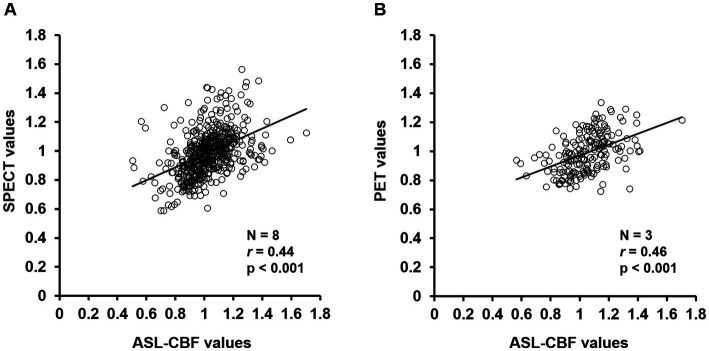
Correlation diagrams of arterial spin labeling (ASL)-cerebral blood flow (CBF) values for single-photon emission computed tomography (SPECT) and positron emission tomography (PET) values in the cerebral cortex regions of all patients with Creutzfeldt–Jakob disease (CJD) **(A,B)**. Regression analysis of the volumes of interest was performed between the normalized mean ASL-CBF values and normalized mean SPECT values **(A)** or PET values **(B)** using a scatter plot with Pearson’s product–moment correlation coefficient. The ASL-CBF values significantly correlated with the SPECT values (*r* = 0.44, *p* < 0.001) and PET values (*r* = 0.46, *p* < 0.001). Pearson correlation coefficients (*r*), value of *p* (p).

The results of the regression analysis showed that the normalized ASL-CBF values significantly correlated with the normalized SPECT (*r* = 0.44, *p* < 0.001) and PET values (*r* = 0.46, *p* < 0.001) in patients with CJD (Figure 6), suggesting that the ASL-CBF values correlated with both the SPECT values and the PET values.

## 4. Discussion

In the present study, ASL-CBF imaging showed quantitative hypoperfusion in cerebral cortical lesions with DWI hyperintensity, similar to ^123^IMP-SPECT and ^18^FDG-PET findings, in patients with CJD. This finding suggests that regional ASL hypoperfusion helps to identify cortical lesions with DWI hyperintensity in patients with CJD, indicating that ASL can precisely detect changes related to CJD.

Recent studies demonstrated that DWI hyperintensity had high sensitivity and specificity in the clinical diagnosis of CJD (9, 31). In particular, the presence of DWI hyperintensity in at least two cortical regions, including the temporal, parietal, and occipital lobes, or both the caudate nucleus and putamen, showed high sensitivity and specificity for the clinical diagnosis of sCJD in patients with dementia disorders (7). Identifying these DWI hyperintensity patterns could distinguish CJD from other non-prion disorders associated with clinically rapid dementia, such as Alzheimer’s disease (32). However, another recent study showed that in 33% (N = 18/55) of patients with sCJD, the diagnosis was delayed because isolated DWI hyperintensity in the cerebral cortex was observed without any concomitant DWI hyperintensity in the basal ganglia, which is contrary to typical findings (33). Since epilepsy and AE also show DWI hyperintensity in the cerebral cortex, some patients with CJD showing isolated DWI hyperintensity in the cerebral cortex might be misdiagnosed with AE or epilepsy. A challenge in the early detection of CJD has been to rule out AE. Among the 384 patients autopsied for clinically suspected CJD, 203 patients were pathologically diagnosed with definite CJD, whereas 181 patients were pathologically diagnosed with other neurodegenerative disorders; in addition, 22 patients of the latter were pathologically diagnosed with AE at autopsy (34). In this report, 14 of these 21 patients (67%) with pathologically proven AE met the U.S. Centers for Disease Control’s diagnostic criteria for sCJD 2010 for possible or probable CJD before their death (34). In another report, central nervous system-specific antibodies were detected in four of the 82 cases (5%) with probable or definite sCJD (35). Conversely, a patient with AE with anti-voltage-gated potassium channel complex antibodies was initially misdiagnosed with CJD due to the presence of DWI hyperintensity in the cortex and CJD-like clinical features (11). These reports indicate the difficulty in differentiating CJD from AE.

As mentioned above, although DWI hyperintensity has been shown to be useful for the diagnostic imaging of CJD, DWI findings alone have limited diagnostic capabilities. Therefore, adding other imaging modalities, such as cerebral perfusion imaging, can improve diagnostic accuracy. In the present study, the mean ASL-CBF values for 70% of the 10 patients with CJD in the DWI-High regions were significantly lower than those in the DWI-Normal regions (Figure 3), whereas none of the 18 HCs and three patients with AE showed differences between the DWI-High regions and DWI-Normal regions (Supplementary Image 2). When visually compared with a patient with AE, a representative patient with CJD showed ASL hypoperfusion in the cerebral cortex consistent with DWI hyperintensities (Figure 2). Visual comparisons of the ASL images suggest that the combination of DWIs and ASL images improves the ability to differentiate AE from CJD.

In a recent case report on the use of ASL imaging, a patient with CJD with the D178N-129 M haplotype in the *PRNP* gene showed severe ASL hypoperfusion and DWI hyperintensity in the cerebral cortex, basal ganglia, and thalamus 6 months after the onset of neurological symptoms (36). In contrast, a patient with AE having lesions with DWI hyperintensity showed regional ASL hyperperfusion in the cerebral cortex (37). In addition, another study found that 10 of 13 patients with status epilepticus exhibited regional ASL hyperperfusion in the cortical territories corresponding to DWI hyperintensity and EEG abnormalities (38). Therefore, the combination of DWI hyperintensity and ASL hypoperfusion may be unique to CJD, and this feature may help differentiate CJD from other disorders with cortical DWI hyperintensity, including AE and epilepsy.

The present study visually and quantitatively demonstrated regional ASL hypoperfusion, ^123^IMP-SPECT hypoperfusion, and ^18^FDG-PET uptake reduction in the cortical lesions showing DWI hyperintensity in patients with CJD. Similar to our findings, a recent case study reported reduced ^18^FDG-PET uptake consistent with both DWI hyperintensity and ASL hypoperfusion in the bilateral cortex, thalamus, and cerebellum in a patient with CJD (39). The present study also showed that the overall agreement rate between DWI and ASL-CBF (81.8%) was similar to that between DWI and ^18^FDG-PET (78.5%) or ^123^IMP-SPECT (85.2%) in patients with CJD (Table 2). Similarly, Xing et al. showed that the overall agreement rate between DWI and ^18^FDG-PET was 74.1% for all cortical regions in 14 patients with CJD (16). Collectively, these results showed that ASL hypoperfusion, ^123^IMP-SPECT hypoperfusion, and ^18^FDG-PET uptake reduction in the cortical lesions showing DWI hyperintensity were significantly consistent.

Notably, the present study showed that the ASL-CBF values significantly correlated with both ^123^IMP-SPECT perfusion values and ^18^FDG-PET uptake values (Figure 6), suggesting that ASL imaging may have a capability similar to that of ^123^IMP-SPECT and ^18^FDG-PET in the detection of regional hypoperfusion or hypometabolism, thereby aiding in the diagnosis of CJD. Furthermore, compared with ^123^IMP-SPECT and ^18^FDG-PET imaging, ASL has several advantages, including a relatively low cost, absence of the need for contrast agent administration, and easy reproducibility. Taken together, our findings suggest that ASL could be a useful noninvasive imaging method for detecting regional hypoperfusion accompanied by DWI hyperintensity in patients with CJD and could be an alternative to ^123^IMP-SPECT and ^18^FDG-PET.

The present study was limited by its single-center, retrospective design. Another possible limitation was that both ^123^IMP-SPECT and ^18^FDG-PET were not performed in all 10 included patients with CJD, the HCs, and the patients with AE. In addition, ASL perfusion technique has potential limitations. ASL tends to show motion artifacts (40), and it remains unclear whether cardiac output and cerebral vascular stenosis affects ASL images (40). The present study ensured that no subjects with significant motion artifacts, severe heart failure, or cerebrovascular stenosis were included. However, the possibility that ASL might not accurately reflect CBF in the basal ganglia and brainstem regions was considered due to potential limitations of the ASL technique. Therefore, the present study evaluated the accuracy of ASL findings in the cortical regions compared to that of SPECT and PET.

In conclusion, ASL-CBF images showed quantitative hypoperfusion in cortical lesions with DWI hyperintensity, similar to ^123^IMP-SPECT and ^18^FDG-PET findings. Moreover, ASL hypoperfusion, ^123^IMP-SPECT hypoperfusion, and ^18^FDG-PET uptake reduction in cortical lesions showing DWI hyperintensity were significantly consistent. ASL-CBF values were significantly correlated with both ^123^IMP-SPECT perfusion and ^18^FDG-PET uptake values. Overall, the present study demonstrated that ASL hypoperfusion consistent with DWI hyperintensity can help distinguish CJD from other disorders with cortical DWI hyperintensity, particularly treatable AE. Compared with ^123^IMP-SPECT and ^18^FDG-PET imaging, ASL imaging has the advantages of feasibility of performing studies as part of standard MRI protocols, including DWIs. Furthermore, the present study demonstrated that ASL can precisely detect changes related to CJD. Therefore, regional cortical hypoperfusion evaluated using ASL imaging could be a useful diagnostic finding in CJD.

## Data availability statement

The raw data supporting the conclusions of this article will be made available by the authors, without undue reservation.

## Ethics statement

The studies involving human participants were approved by the Institutional Review Board of the University of Fukui (20170130). The studies were conducted in accordance with the local legislation and institutional requirements. The ethics committee/institutional review board waived the requirement of written informed consent for participation from the participants or the participants’ legal guardians/next of kin because of the retrospective nature of this study.

## Author contributions

YK: writing the manuscript. MI: design of the research hypothesis and study, interpretation of the study results, and critical review of the manuscript. TH: conception, design, writing, and revising the manuscript. HS, TY, SE, NS, KH, and OY: design of the research hypothesis and study, interpretation of the study results, and critical review of the manuscript. TT, HO, and HK: design of the research hypothesis and study, interpretation of the imaging results, and critical review of the manuscript. YN: initial concept and design of the research hypothesis and study, interpretation of the study results, and critical review of the manuscript. All authors contributed to the study conception and design, read and approved the final manuscript.

## Abbreviations

ASL: arterial spin labeling
CJD: Creutzfeldt–Jakob disease
HCs: healthy controls
PLD: post-labeling delay
PSDs: periodic synchronous discharges
RT-QUIC: real-time quake
sCJD: sporadic type of CJD
VOIs: volumes of interest
