# Supplementary material for: Magnetic resonance imaging arterial spin labeling hypoperfusion with diffusion-weighted image hyperintensity is useful for diagnostic imaging of Creutzfeldt–Jakob disease
Source: Front Neurol. 2023 Oct 10;14:1242615. doi: 10.3389/fneur.2023.1242615 (PMC10598551; doi:10.3389/fneur.2023.1242615)
Supplement: Supplementary file 1 [file Table_1.pdf]

Supplementary Table 1. Comparison between the mean and standard deviation of the normalized values of ASL-CBF, SPECT, and PET in the DWI-High and DWI-Normal regions in patients with CJD.

CJD patient #	ASL-CBF values (N = 10)		SPECT values (N = 8)		PET values (N = 3)	
	DWI-High	DWI-Normal	DWI-High	DWI-Normal	DWI-High	DWI-Normal
CJD 1	0.92 ± 0.10 [c]	1.07 ± 0.14	0.89 ± 0.06 [d]	1.00 ± 0.12	0.89 ± 0.08 [d]	1.00 ± 0.11
CJD 2	0.91 ± 0.08 [b]	1.03 ± 0.10	0.85 ± 0.08 [a]	1.04 ± 0.13	0.88 ± 0.13 [c]	1.03 ± 0.15
CJD 3	0.93 ± 0.23 [d]	1.11 ± 0.20	0.90 ± 0.14	0.96 ± 0.16	0.92 ± 0.11	0.99 ± 0.14
CJD 4	0.75 ± 0.10 [a]	1.11 ± 0.14	0.76 ± 0.10 [a]	1.05 ± 0.14	N/A	N/A
CJD 5	0.87 ± 0.09 [d]	1.02 ± 0.16	0.84 ± 0.17	0.98 ± 0.20	N/A	N/A
CJD 6	0.97 ± 0.09	1.00 ± 0.11	0.86 ± 0.09 [d]	1.00 ± 0.18	N/A	N/A
CJD 7	0.98 ± 0.12	1.00 ± 0.09	0.92 ± 0.09	1.00 ± 0.15	N/A	N/A
CJD 8	1.01 ± 0.13	1.00 ± 0.16	0.99 ± 0.09	1.01 ± 0.13	N/A	N/A
CJD 9	0.92 ± 0.11 [b]	1.14 ± 0.21	N/A	N/A	N/A	N/A
CJD 10	0.87 ± 0.13 [a]	1.17 ± 0.23	N/A	N/A	N/A	N/A
CJD mean	0.91 ± 0.15 [a]	1.06 ± 0.17	0.88 ± 0.12 [a]	1.01 ± 0.16	0.90 ± 0.11 [a]	1.00 ± 0.13

t-test was used to evaluate the differences in the normalized ASL-CBF values between the DWI-High and DWI-Normal regions. ASL; Arterial spin labeling, CBF; Cerebral blood flow, CJD; Creutzfeldt–Jacob disease, DWI; Diffusion–weighted image, HC; Healthy control, M; Male, N/A; Not available, PET; Positron emission tomography, SPECT; Single-photon emission computed tomography, VOI; Volume of interest. [a] p-value of t-test < 0.001, [b] p-value of t-test < 0.005, [c] p-value of t-test < 0.01, [d] p-value of t-test < 0.05, compared with DWI-normal.
