# Supplementary figures and images for: Magnetic resonance imaging arterial spin labeling hypoperfusion with diffusion-weighted image hyperintensity is useful for diagnostic imaging of Creutzfeldt–Jakob disease
Source: Front Neurol. 2023 Oct 10;14:1242615. doi: 10.3389/fneur.2023.1242615 (PMC10598551; doi:10.3389/fneur.2023.1242615)

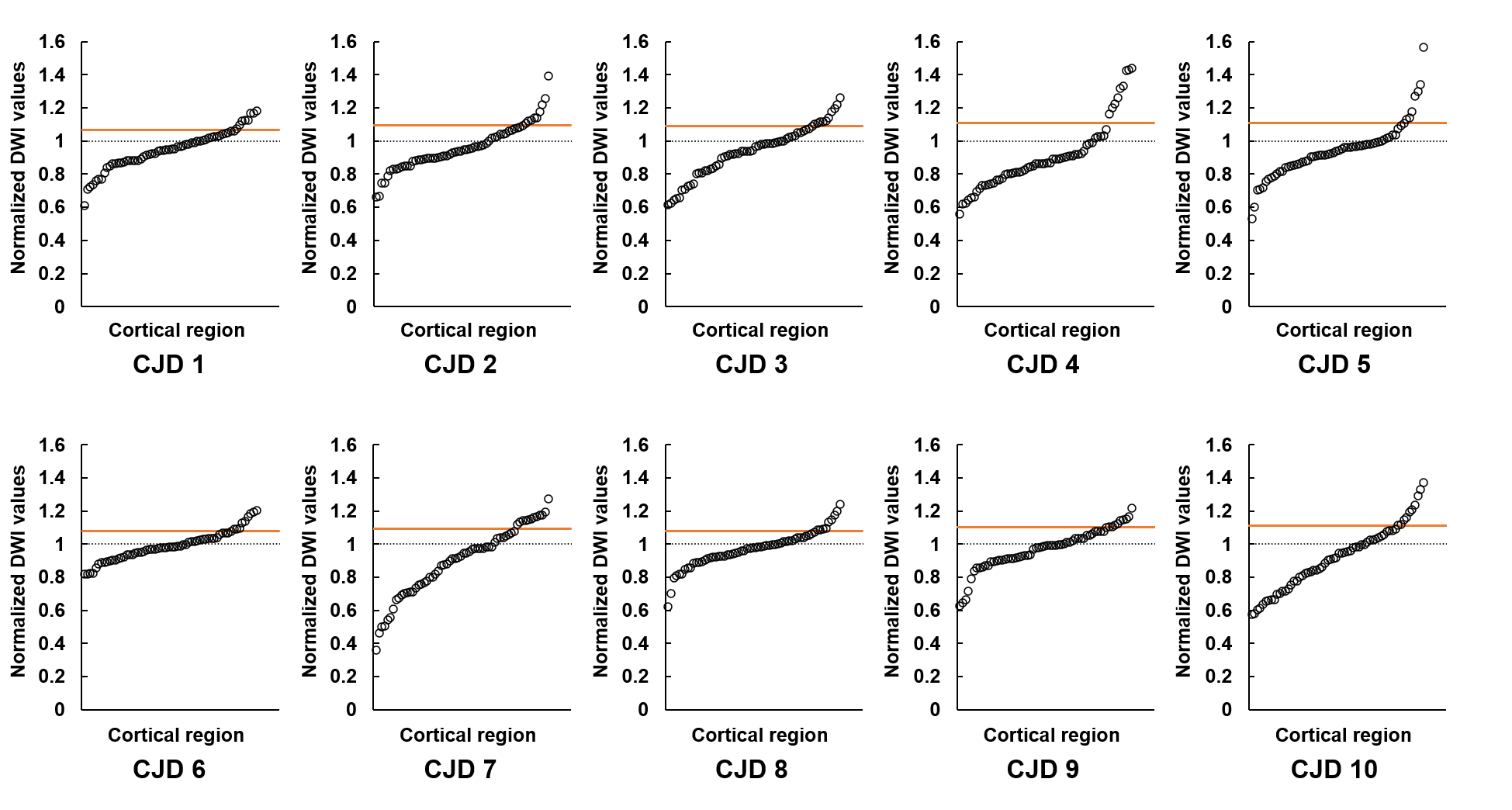

Supplement: Supplementary file 3 [file Image_1.TIF]

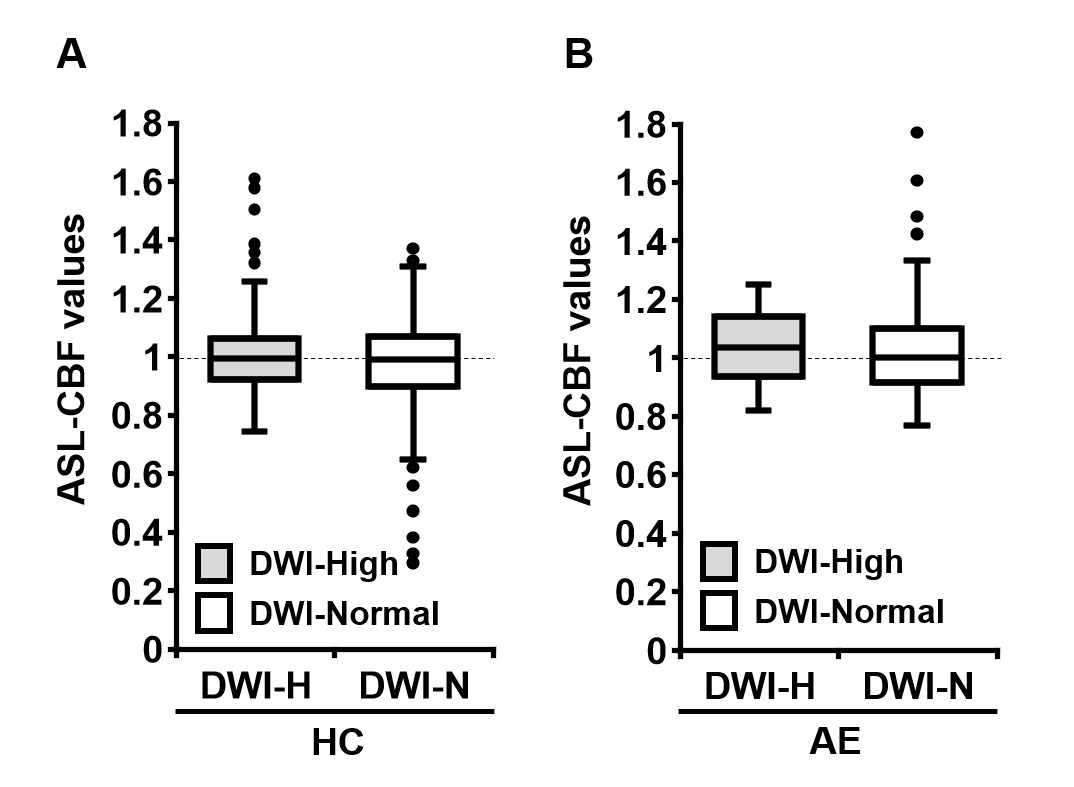

Supplement: Supplementary file 4 [file Image_2.TIF]
